# Case Report: Amyloidosis Cutis Dyschromica: Dermoscopy and Reflectance Confocal Microscopy and Gene Mutation Analysis of a Chinese Pedigree

**DOI:** 10.3389/fmed.2021.774266

**Published:** 2021-12-01

**Authors:** Hui Wang, Zhenyu Zhong, Xiuli Wang, Liyun Zheng, Yifan Wang, Shan Wang, Siqi Liu, Hui Li, Ze Guo, Min Gao

**Affiliations:** ^1^Department of Dermatology, No. 1 Hospital, Anhui Medical University, Hefei, China; ^2^Institute of Dermatology, Anhui Medical University, Hefei, China; ^3^Key Laboratory of Dermatology, Ministry of Education, Anhui Medical University, Hefei, China; ^4^Anhui Provincial Institute of Translational Medicine, Hefei, China; ^5^Inflammation and Immune Mediated Diseases Laboratory of Anhui Province, Anhui, China

**Keywords:** amyloidosis cutis dyschromica, dermoscopy, reflectance confocal microscopy, non-invasive techniques, mutation

## Abstract

**Background:** Amyloidosis cutis dyschromica (ACD) is a rare type of primary localized cutaneous amyloidosis. Non-invasive techniques can provide important clues for early diagnosis.

**Objectives:** To highlight the characteristic imaging changes of ACD under dermoscopy and reflectance confocal microscopy (RCM), investigate gene mutations in a Chinese Han pedigree of ACD, and analyze the genotype–phenotype correlation.

**Methods:** Dermoscopy and RCM examinations were completed together for the pedigree, and the imaging characteristics were described. The diagnosis of ACD was confirmed by pathological examination. Sequencing was performed followed by bioinformatics and genotype–phenotype correlation. ACD-related articles published on PubMed between January 1970 and March 2021 were reviewed and summarized.

**Results:** In ACD, dermoscopy showed patchy white hypopigmentation and brownish spots, stripes, or hyperpigmented blotches and patches. RCM showed a highly refractive substance with clumpy, dotted, and linear structures inside the papillary dermis. Sequencing identified glycoprotein non-metastatic melanoma protein B (*GPNMB*) missense mutations [c.393T>G (p.Y131X; NM_001005340.2)] and a frameshift deletion mutation [c.719_720delTG (p.V240fs; NM_001005340.2)]. The ANNOtate VARiation (ANNOVAR) software predicted that c.393T>G is a pathogenic mutation. The literature review found 14 mutations, namely, 5 (35.7%) frameshift mutations, 4 (28.6%) non-sense mutations, 4 (28.6%) missense mutations, and 1 (7.1%) splice site mutation. Blisters and epidermolysis were observed in several cases, but there was no significant association between clinical manifestations and mutations in ACD.

**Conclusions:** This study was the first to combine dermoscopy and RCM to describe ACD. Two *GPNMB* gene mutations were reported in a Chinese ACD pedigree. The genotype–phenotype correlation was analyzed for the first time; however, there was no significant correlation.

## Introduction

Primary localized cutaneous amyloidosis (PLCA) is a group of chronic pruritic skin diseases characterized by amyloid deposits in the upper dermis. Amyloidosis cutis dyschromica (ACD) is a rare type of PLCA first described by Morishima in 1970 (1). In 2018, mutations of the glycoprotein non-metastatic melanoma protein B (*GPNMB*) were proven to be associated with ACD (2). Later, additional mutation sites on *GPNMB* related to ACD were identified.

Providing evidence through imaging is important for early diagnosis. However, so far, there have only been few reports concerning the imaging of ACD (3, 4), and no article has provided a unified description of the imaging characteristics.

To our knowledge, this is the first study to combine dermoscopy, reflectance confocal microscopy (RCM), and pathology to describe the features of ACD, aimed at helping clinicians establish a diagnosis and perform timely interventional measures. Furthermore, we performed sequencing, bioinformatic, and genotype–phenotype correlation analysis on a Chinese Han pedigree.

## Materials and Methods

This study was performed in adherence with the principles of the Declaration of Helsinki and was approved by the Ethical Review Committee of Anhui Medical University. A Chinese Han family diagnosed with ACD was recruited from the First Affiliated Hospital of Anhui Medical University (Hefei, China). All participants signed informed consent forms.

Clinical images were taken using a digital camera. Punch biopsy specimens were obtained from back skin lesions. Paraffin sections were prepared, stained with hematoxylin and eosin and Congo red (CR), and examined by light microscopy. Examinations were performed using both dermoscopy and RCM. All dermoscopic (Dermoscopy-II; Dermat®, Beijing, China) and RCM (Vivascope 3,000® Lucid Inc., Rochester, NY, United States) images were captured appropriately and analyzed.

Peripheral blood (3–5 ml) was collected from each patient. Genomic DNA was extracted from peripheral blood lymphocytes using Qiagen (No: 51206; Qiagen, Hilden, Germany) genomic DNA extraction kits and stored at −80°C. Qualified genomic DNA samples were analyzed by Sanger sequencing. The results were analyzed using the DNA Sequencing analysis software. Published data were collected to conduct an association analysis between genotype and phenotype.

## Results

**Figure 1:**
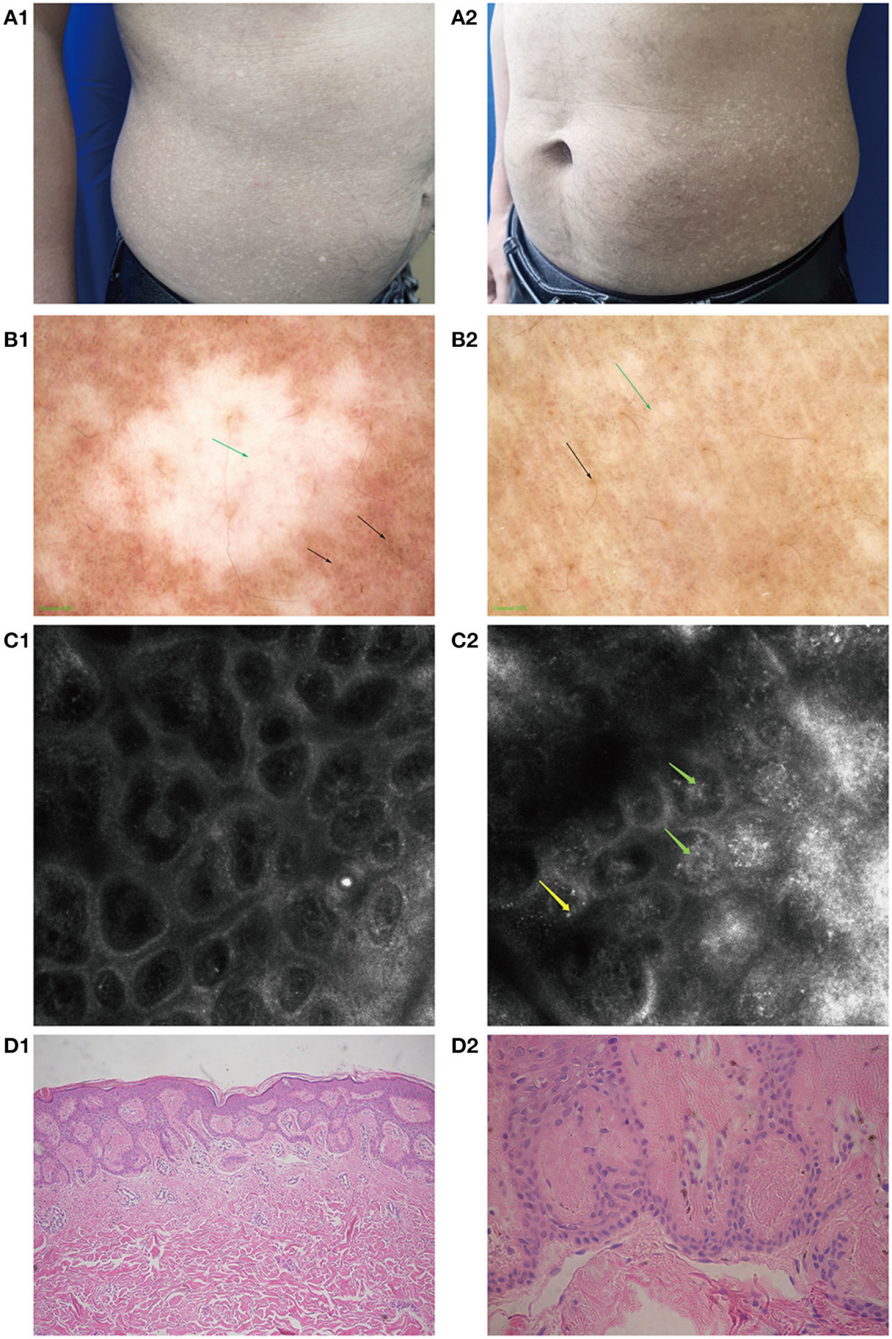
Clinical manifestations, imaging changes, and pathological changes in amyloidosis cutis dyschromica (ACD). **(A1,A2)** Hyperpigmented and hypopigmented macules on the abdomen of a patient with ACD. **(B1,B2)** Feature of ACD under dermoscopy. **(B1)** Hypopigmented areas: white patch (green arrow) with blurred margins, white and brown blotches (black arrow) surrounded. **(B2)** Hyperpigmented areas: large fulvous pigmentation spots (black arrow), interspersed with white grid-like stripes and patches (green arrow). **(C1)** RCM performance in some normal skin areas: no obvious amorphous deposits. **(C2)** Feature of ACD under RCM. The highly refractive substance inside the papillary dermis (like clumpy, dotted, and linear structures, green arrow) and highly refractive round cell scattered in the superficial dermis (yellow arrow). **(D1)** Mildly hyperkeratotic epidermis along with amorphous eosinophilic materials in the papillary dermis (HE, ×100). **(D2)** Eosinophilic materials stained positive (Congo red, ×400).

The proband was a 35-year-old man with dense hyperpigmentation and hypopigmented spots on the trunk and limbs. He has had progressive, asymptomatic, diffused pathological changes since the age of 8 years old. At first, hyperpigmentation appeared on his back; but gradually, the abdomen, limbs, and palmoplantar regions also became affected. He did not have a history of systemic diseases or other skin diseases (Figure 1A1,A2). His 14-year-old son and 5-year-old daughter had no symptoms at the time of this study. Similar skin lesions were also present in his 32-year-old brother.

Under dermoscopy, the hypopigmented areas showed big white patches with poorly defined margins, surrounded by small white spots and hyperpigmented blotches and patches. The hyperpigmented areas were patches composed of brownish spots, intermixed with striped or patchy white areas of hypopigmentation (Figure 1B1,B2).

The features of ACD, as seen under RCM, were hyperkeratosis in the epidermis: a highly refractive substance with clumpy, dotted, and linear structures inside the papillary dermis, and widening of the ring structure of the dermal papilla. Parts of the hypopigmented areas showed highly refractive round cells scattered in the superficial dermis (Figure 1C2).

The patient underwent a biopsy examination. Histopathology revealed sparse melanophages and focal pigment incontinence beneath the epidermis, mild hyperkeratosis in parts of lesions, and homogenous eosinophilic deposits in the papillary dermis that stained positive with CR and was consistent with amyloidosis (Figure 1D1,D2).

**Figure 2:**
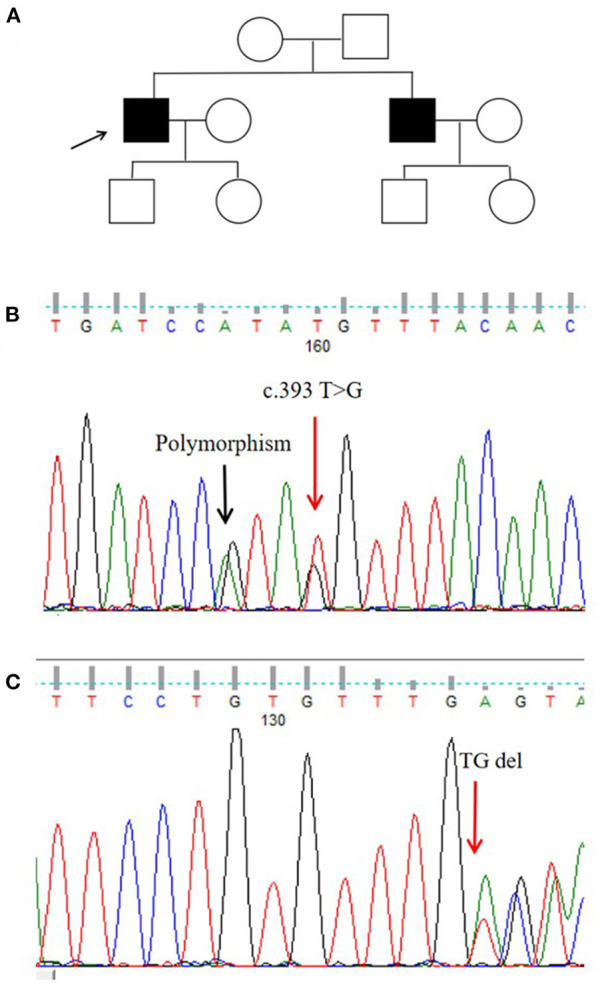
Pedigree map and gene mutation sites. **(A)** Pedigree of the proband. **(B,C)** Glycoprotein non-metastatic melanoma protein B (GPNMB) mutations detected in the proband.

We identified a missense mutation (c.393T>G; NM_−_001005340.2) and a frameshift mutation (c.719_−_720delTG; NM_−_001005340.2). The ANNOVAR annotation software indicated that the frequency of c.393T>G in all populations was 0.00001648, suggesting that this missense mutation was a pathogenic mutation (Figures 2B,C).

**Figure 3:**
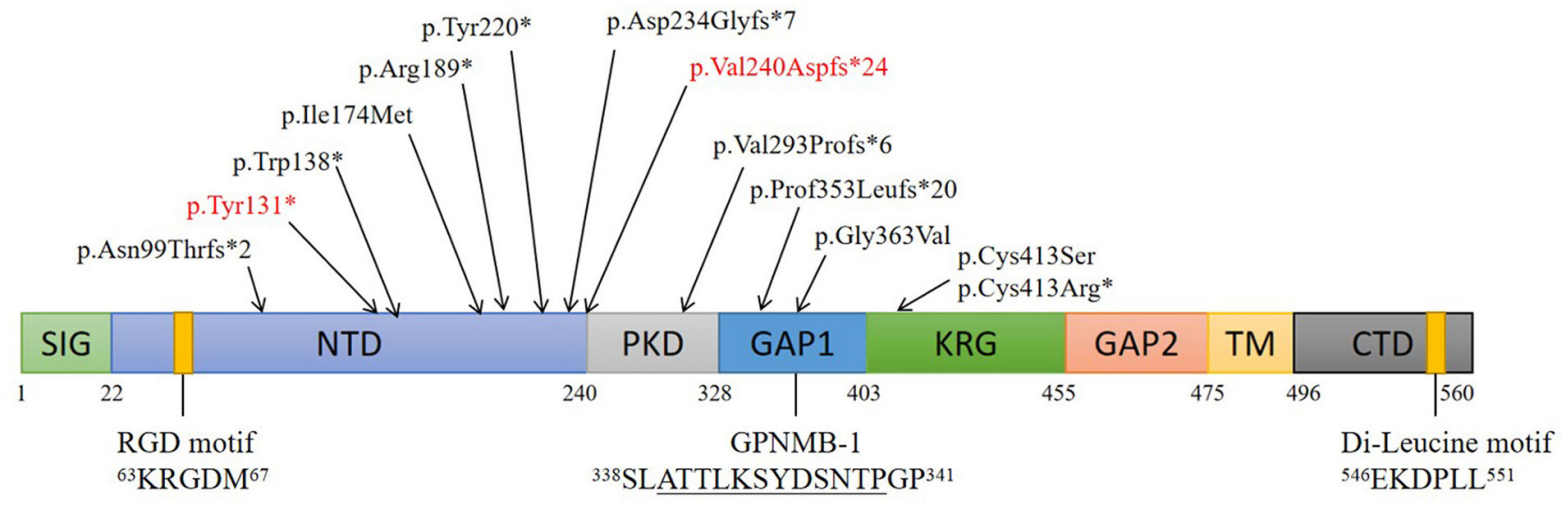
Structure and mutations of GPNMB. Schematic of 8 domains on human GPNMB and gene mutations responsible for ACD. Numbers correspond to amino acid residues; The RGD and di-leucine motifs are shown. A splice isoform of GPNMB (GPNMB-1) with an in-frame 12-amino-acid insertion (underlined) is also shown. Locations of GPNMB mutations (on the protein) identified in this study and those from an earlier study are indicated. (SIG, signal sequence; NTD, N-terminal domain; PKD, polycystic kidney disease-like domain; KRG, kringle-like domain; TM, transmembrane domain; CTD, C-terminal cytoplasmic domain).

Articles published on PubMed from January 1970 to March 2021 were reviewed. About 70 cases of ACD were reported, most of which involved Asian patients. So far, 14 ACD-related gene loci have been found on *GPNMB*, namely, 5 (35.7%) frameshift mutations, 4 (28.6%) non-sense mutations, 4 (28.6%) missense mutations, and 1 (7.1%) splice site mutation (Figure 3).

The data related to gene mutation and clinical manifestation are summarized in Table 1 (2, 5–8). To our best knowledge, there has been no analysis yet of genotype–phenotype correlation in ACD. Blisters and epidermolysis are rare clinical manifestations. Given the summarized results, we investigated the genotype–phenotype correlation; however, no significant association was found.

**Table 1:** Amyloidosis cutis dyschromica (ACD)-related gene mutations reported in the literature published in PubMed from January 1970 to March 2021.

**Mutation**	**Protein**	**Effect on *GPNMB* mRNA**	**Clinical features**		**References**
			**Sites of skin lesion**	**Blisters or other manifestations**	
c.565C>T	p.Arg189^*^	Nonsense	Mainly on the neck, trunk, and limbs	Blisters	(2)
c.660T>G	p.Tyr220^*^	Nonsense	Neck, trunk, and limbs	Blisters	
c.296delA	p.Asn99Thrfs^*^2	Frameshift	Neck, trunk, and limbs	No	
c.719_720delTG	p.Val240Aspfs^*^24	Frameshift	Face, neck, trunk, limb	No	
c.877_880delGTTT	p.Val293Profs^*^6	Frameshift	Face, neck, trunk, limb	No	
c.1056delT	p.Pro353Leufs^*^20	Frameshift	Neck, trunk, limbs, hands, feet	No	
c.700+5G>T	p.Asp234Glyfs^*^7	Splice site	Not reported	Not reported	(5)
c.1238G>C	p.Cys413Ser	Missense	Not reported	Not reported	
c.393T>G	p.Tyr131^*^	Nonsense	All the body except for the face, palms and soles	Blisters and epidermolysis	(6)
c.413G>A	p.Trp138^*^	Nonsense	All the body except for the face, palms and soles	Blisters and epidermolysis	
c.717_718delTG	p.Val240Aspfs^*^24	Frameshift	All the body except for the face, palms and soles	Blisters and epidermolysis	
c.1237T>C	p.Cys413Arg^*^	Missense	Trunk and limbs	No	(7)
c.1088G>T	p.Gly363Val	Missense	Trunk, limbs, neck	No	(8)
c.522C>G	p.Ile174Met	Missense	Trunk, limbs, neck	No	

* * means terminator,one of the amino acid naming rules*.

## Discussion

Amyloidosis cutis dyschromica is a special type of PLCA that often begins before puberty. The main manifestations are reticular and dotted pigmentation with hypopigmented spots that can spread all over the body, with or without itching. Some uncommon manifestations have also been reported, such as blisters (2, 9) and lichenoid papules (10).

In this Chinese Han pedigree, hyperpigmentation spots and hypopigmentation spots were densely distributed on the trunk and limbs of the proband. There were no other abnormalities, such as pruritus, photosensitivity, or blisters. The patient developed symptoms at the age of 8 years, and his brother had a similar manifestation. His parents were not close relatives (Figure 2A).

In 2016, Mahon et al. (11) reported that ACD was sporadic and familial. The average age of onset of familial cases is 6 years, whereas the average age of onset of sporadic cases is 23 years. The time from the onset to diagnosis ranges from 20 to 40 years. The lag of diagnosis suggests that more effective diagnostic methods are needed for ACD.

The value of the combination of dermoscopy and RCM has been confirmed in the diagnosis of diseases such as primary cutaneous amyloidosis and keratoacanthoma (12, 13).

In 2009, Ye et al. (3) described the characteristics of ACD under RCM as a highly refractive substance deposit with significant telangiectasia in the papillary dermis, which is consistent with our results. RCM showed clumpy, dotted, and linear collections of a highly refractive substance in the papillary dermis. In 2018, Wang et al. (4) described the characteristics of ACD under dermoscopy as unified spots and pigmentation in different lesions. Changes in hypopigmented and hyperpigmented areas were highlighted separately in this study. We combined RCM, dermoscopy, and histopathology for the first time, specifically highlighting the imaging characteristics of ACD, which would provide a basis for diagnosis and timely intervention.

We correlated the characteristics of skin imaging with histologic findings. Our histopathological results show mild hyperkeratosis, focal pigment incontinence, and homogenous eosinophilic deposits in the papillary dermis. Based on these typical pathological characteristics (14), the diagnosis of ACD was confirmed. The literature shows that amyloid deposits in PLCA correspond to various high refractive structures under RCM, usually with various shapes (dotted, coliform, and agglomerates), and melanophages, often visible as brightly refractile, oval or stellate-shaped cells. In addition, characteristics such as increased melanin in the basal layer of the epidermis, mildly acanthoid epidermis, and slight hyperkeratosis can be enhanced under RCM (15). These advantages are also reflected in ACD. Lei et al. suggested that the correspondence between high refractive index in RCM images and amyloid deposition in dermal papillae is specific, which helps the RCM evaluation of PLCA, this conclusion was summarized by lichen amyloidosus and macular amyloidosis. Our study on ACD, a rare type of PLCA, also has this characteristic (16).

Qiao et al. found that patients with ACD have similar protein profiles, with strong positive expression of cytokeratin markers *CK334*β*E12* and *CK5/6*. This finding revealed that the amyloid of ACD was derived from keratinocytes (17). *In vitro*, it has been shown that *GPNMB* knockdown in melanocytes induced keratinocyte apoptosis (5). *GPNMB*, a highly glycosylated type I transmembrane glycoprotein that is homologous with *PMEL17*, plays an important role in inflammation regulation and is involved in the pathogenesis of ACD (2).

At present, 14 *GPNMB* gene mutations associated with ACD have been identified. Among these, five (35.7%) are frameshift, four (28.6%) are non-sense, four (28.6%) are missense, and 1 (7.1%) is a splice site mutation. In addition, seven (50%) mutations are in the N-terminal domain (NTD), which suggests that *GPNMB* mutations play a critical role in ACD, and that NTD is an important functional region on *GPNMB* related to the etiology of ACD.

In this study, we investigated a Chinese Han ACD pedigree. Sanger sequencing revealed a c.393T>G mutation, leading to the corresponding amino acid being changed from tyrosine to other amino acids (p.Y131X). Another mutation, a 2-bp c.719_720delTG deletion, results in a frameshift after the 240th valine (p.V240fs). These two mutations were reported in different pedigrees (2, 6).

Although patients with ACD have similar clinical features, blisters and epidermolysis are rarely observed (2). Some scholars believe that a few clinical features are overlapping between ACD and other types of PLCA, such as vesicles (2). Analyzing the genotype–phenotype correlation of ACD, we hypothesized that the blisters could be associated with some gene mutations such as c.565C>T and c.660T>G. However, no significant relevance was found. This conclusion could be a result of the small sample size.

There is also a hypothesis that damage to the DNA repair mechanism induced by ultraviolet B and ultraviolet C will lead to repeated damage and even apoptosis of keratinocytes. This damage has potential genetic susceptibility and is a possible pathogenic factor (18, 19).

There is currently no unified treatment plan for ACD. Treatment methods based on reports include topical retinoic acid, keratolytics, steroids, capsaicin, and carbon dioxide laser (20). Topical treatment (10% urea cream and tazarotene), oral vitamins, and antioxidants are not effective (11). Some cases suggest that acitretin therapy can improve skin symptoms (11, 18, 21).

In summary, to our knowledge, this is the first combination of dermoscopy and RCM to describe ACD. This is also the first study to analyze genotype–phenotype correlation in patients with ACD. Recognizing the characteristic changes under dermoscopy and RCM provides the possibility of early diagnosis of ACD; however, further studies with larger samples are needed to verify the genotype–phenotype correlation in ACD.

## Data Availability Statement

The original contributions presented in the study are included in the article/supplementary material, further inquiries can be directed to the corresponding author/s.

## Ethics Statement

The studies involving human participants were reviewed and approved by Ethical Review Committee of Anhui Medical University. The patients/participants provided their written informed consent to participate in this study. Written informed consent was obtained from the individual(s) for the publication of any potentially identifiable images or data included in this article.

## Author Contributions

All authors listed have made a substantial, direct, and intellectual contribution to the work and approved it for publication.

## Conflict of Interest

The authors declare that the research was conducted in the absence of any commercial or financial relationships that could be construed as a potential conflict of interest.

## Publisher's Note

All claims expressed in this article are solely those of the authors and do not necessarily represent those of their affiliated organizations, or those of the publisher, the editors and the reviewers. Any product that may be evaluated in this article, or claim that may be made by its manufacturer, is not guaranteed or endorsed by the publisher.
